# Thermal stress responses and heat stress resilience genes in chickens are revealed through genomic and transcriptomic insights

**DOI:** 10.1186/s40104-025-01283-w

**Published:** 2026-03-09

**Authors:** Md Mortuza Hossain, Jinhyun Ahn, Soo-Youn Choi, Sung-Pyo Hur, Dajeong Lim, Donghyun Shin, Sanghoon Lee, Jong-Eun Park

**Affiliations:** 1 Department of Animal Biotechnology, College of Applied Life Science, Jeju National University, Jeju-si, Republic of Korea; 2 Department of Management Information Systems, Jeju National University, Jeju-si, Republic of Korea; 3 Department of Biology, Jeju National University, Jeju-si, Republic of Korea; 4 Department of Marine Life Science, Jeju National University, Jeju-si, Republic of Korea; 5 Department of Animal Resources Science, Chungnam National University, Daejeon, Republic of Korea; 6 Department of Agricultural Convergence Technology, Jeonbuk National University, Jeonju, Republic of Korea

**Keywords:** Genetic adaptation, Heat stress, Poultry thermotolerance, Thermal stress, Transcriptomics

## Abstract

Climate change creates major challenges in livestock industry, making chickens vulnerable to heat stress because they can tolerate a narrow range of temperatures. Heat stress disrupts metabolic and physiological homeostasis, leading to reduced growth, productivity, reproduction, and immune function, thereby threatening the economic viability of poultry farming. This review explores the multifaceted impacts of heat stress on poultry, including physiological responses, production performance, and immune function. Recent advances in transcriptomic and genomic research have shed light on the molecular mechanisms underlying heat stress resilience in poultry. Key genes such as *HSP70*, *HSP90*, *HSP27*, and *HSP47* are significantly upregulated under heat stress, playing vital roles in protein folding, preventing aggregation, and protecting cellular integrity. Additionally, genes like *SOD* and *CAT* enhance antioxidant defenses, mitigating oxidative damage. Genes such as *RB1CC1*, *BAG3*, and *TRMT1L* regulate apoptosis and oxidative stress, promoting cell survival. In the liver, CCK, *DIO3*, and *ANGPTL4* improve energy homeostasis and reduce metabolism-related heat production, while *BMP10* and *MYH7* in the heart contribute to cardiac adaptation during thermal stress. Genetic adaptations such as the Naked neck, Frizzle, and Dwarf gene provide intrinsic thermotolerance by reducing feather mass, altering feather structure, and minimizing body size, thereby improving heat dissipation. These genetic traits, combined with transcriptomic insights into heat resilience genes, offer opportunities for developing heat-tolerant chicken breeds. By integrating molecular genetics, transcriptomics, and management strategies, this review highlights the importance of selective breeding programs to enhance poultry thermotolerance. Future research should focus on leveraging indigenous breeds, advanced molecular tools, and nutritional interventions to mitigate the effects of rising global temperatures. Enhancing heat stress resilience in poultry is imperative to ensure sustainable production and global food security in this climate change.

**Graphical Abstract:**

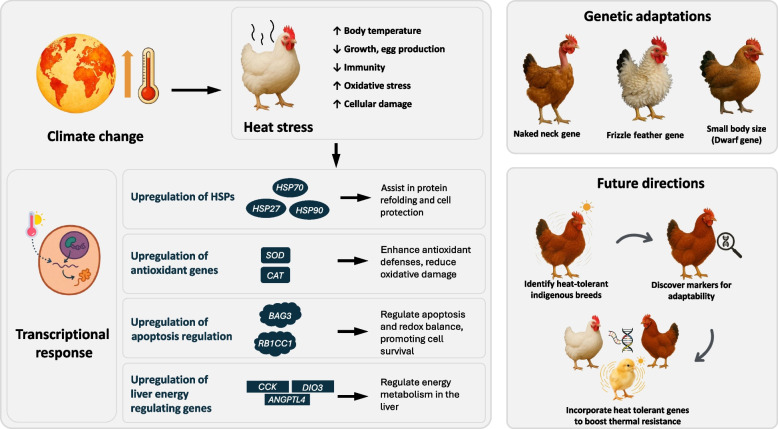

## Introduction

Climate change has emerged as a significant disruption in agricultural systems, presenting livestock production with challenges never faced before. Among livestock, poultry industry plays a vital role in global agriculture, providing a primary source of protein for billions of people worldwide. However, the resilience of chicken production to climatic stressors, particularly heat stress has emerged as a critical concern for scientists and poultry producers [1, 2]. As chickens are homeothermic, they maintain a constant body temperature through a delicate balance of metabolic heat production and heat loss [3]. However, this balance is severely disrupted under conditions of extreme heat, leading to a condition known as heat stress. Heat stress not only compromises welfare but also leads to significant reductions in productivity, including decreased growth rates, egg production, and meat quality, thereby posing a substantial threat to the economic viability of poultry farming [2]. Recent advances in genomic technologies have enabled an unprecedented exploration of the molecular mechanisms underlying heat stress resilience in chickens. High-throughput sequencing technologies, such as ribonucleic acid (RNA) sequencing, have been instrumental in identifying key genes and pathways implicated in the heat stress response [4]. The transcriptomic response of chickens to heat stress involves a complex network of gene expression changes [4]. These changes underline the physiological and metabolic adaptations that chickens employ to cope with heat stress. Among the most crucial molecular players in this response are heat shock protein (HSP), which facilitate protein folding and protect cells from stress-induced damage [3]. Additionally, genes involved in apoptosis, oxidative stress pathways, cell proliferation, and immune response are differentially expressed in chickens exposed to high temperatures [1, 5].

Transcriptomic response can influence gene expression offering a dynamic means of adapting to environmental stressors. Investigating these modifications can provide insights into how chickens acclimate to heat stress over their lifespan and across generations. The implications of climate change on poultry production underscore the urgency for research focused on understanding and improving heat stress resilience. This review aims to synthesize the current body of knowledge on the transcriptomic mechanisms of adaptation in chickens, with a particular focus on the study of climate change resilience.

## Heat stress

Heat stress is a significant challenge in poultry production, exacerbated by global warming and the increasing frequency of heat waves. It occurs when ambient temperatures exceed the thermo-neutral zone (16–25 °C) or their ability to dissipate excess heat, leading to physiological disruptions and economic losses [6]. Broilers lack sweat glands, so they rely on physiological and behavioral adaptations to manage heat [7]. These include panting, increasing water intake, reducing feed consumption, and engaging in behaviors such as wing spreading and staying in shaded areas. However, these compensatory mechanisms are often insufficient under extreme or prolonged heat exposure, resulting in reduced production performance, altered metabolism, poor meat and egg quality, dehydration, and increased morbidity and mortality. For broilers, heat stress can lead to excessive fat deposition and reduced muscle mass, while laying hens may experience decreased egg production and compromised egg quality [2]. Different HSP genes, particularly *HSP70* and *HSP90*, play critical roles in protecting cellular structures during heat stress by stabilizing proteins and preventing aggregation [8, 9]. The expression of HSP genes, such as *HSP70*, *HSP90*, and *HSP27*, is time- and tissue-dependent, with acute heat stress inducing rapid upregulation, especially in the heart, liver, and intestines [10]. Epigenetic modifications, such as deoxyribonucleic acid (DNA) methylation of the *HSP70* promoter, have been shown to enhance thermotolerance, particularly in embryos subjected to thermal manipulation [11]. Despite these adaptive responses, heat stress adversely affects poultry health, reducing fertility, production efficiency, and meat quality.

## Mechanisms of body heat regulation

**Fig. 1:**
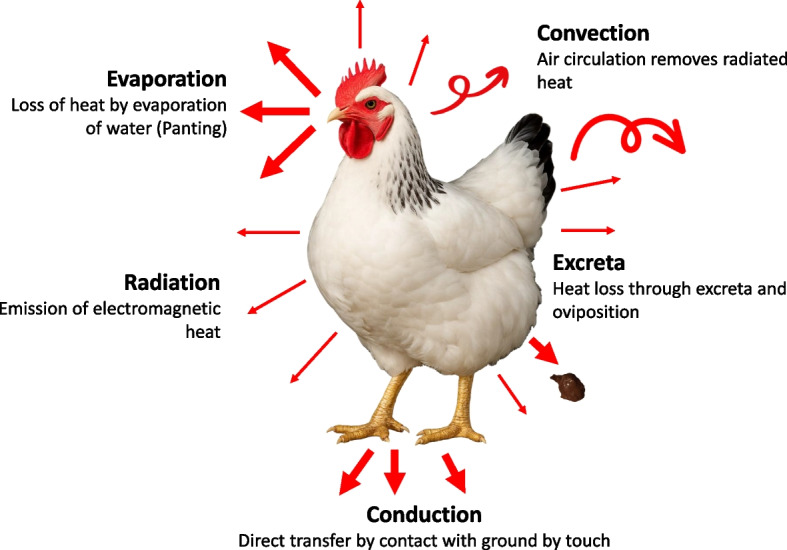
Heat dissipation mechanisms in poultry

Modern poultry genetics and nutrition have increased productivity of commercial poultry such as, laying hens can now lay more than 320 eggs in their first year and broilers can reach a market weight of 2.5–3.0 kg within 35–42 d. These improvements have also increased metabolic activity which leads to more heat production and makes chicken more sensitive to high temperatures. As chicken is a homeothermic animal, they maintain a stable body temperature using five main mechanisms (Fig. 1) [3]. Convection is the most efficient means of heat dissipation where air movement across the body facilitates cooling [12]. This often requires ventilation systems. Radiation allows chicken to lose heat through energy waves to cooler surfaces. This works only when the internal temperature of chicken is higher than the environment. Conduction is heating loss through contact with cooler surfaces. However, it is usually minimal and not very effective. These three methods only work when temperatures are below or within the thermo-neutral zone. At higher temperatures, evaporation becomes the main way chicken loses heat. Panting releases heat by evaporating water from their respiratory tract [7]. Evaporation is less effective in high humidity. Excretion also helps with heat regulation. Birds drink more water and produce wetter excreta to cool down [3]. These mechanisms are vital to helping chickens maintain their body temperature during heat stress.

## Stress-induced neuroendocrine activation

### The sympatho-adrenal medullary pathway

The sympatho-adrenal medullary (SAM) pathway is a key component of the stress response system in chicken. This is responsible for the rapid release of catecholamines, primarily norepinephrine and epinephrine, triggered by activation through stressors [13]. These hormones play a vital role in regulating essential physiological functions, including cardiovascular activity, energy metabolism, and respiratory efficiency. Stressors such as elevated surrounding temperatures, social engagements and livestock management methods can stimulate this pathway, leading to the secretion of catecholamines to mitigate the immediate impact of stress [14, 15].

The SAM pathway is particularly critical in facilitating the "fight-or-flight" response, enabling chicken to adapt quickly to acute stress conditions [16]. Catecholamines influence muscle contraction and relaxation, optimize oxygen delivery by inducing bronchodilation, and modulate energy metabolism to meet the heightened demands imposed by stress [17]. These physiological adjustments are vital for short-term survival during stressed conditions. Interestingly, changes in the levels and ratios of epinephrine and norepinephrine in an animal's body have been utilized as biomarkers to assess stress and overall well-being. In poultry, such hormonal shifts provide insights into the severity of heat stress experienced by the flock [18]. However, while these indicators are useful, they come with certain limitations. For example, the process of blood sampling itself can induce handling stress, potentially altering catecholamine levels and confounding the results. Moreover, individual variability and environmental factors further complicate the interpretation of these measurements.

### The hypothalamic-pituitary-adrenal axis

The hypothalamic-pituitary-adrenal (HPA) axis is integral to an organism's capability to adapt to various stressors, including heat stress [16]. This axis regulates a wide range of physiological responses that are critical for maintaining homeostasis, such as reproductive function, metabolic activity, productivity, and behavior [19]. By modulating hormone secretion, the HPA axis ensures that the body can respond dynamically to environmental changes while preserving internal balance. Interestingly, heightened activity in the HPA axis does not exclusively correspond to stress-related events. For example, cortisol levels can increase naturally during postprandial periods (after eating) or in response to physical exertion, underscoring the complex and multifunctional role of this system in physiology [20, 21].

In poultry, as in other vertebrates, the adrenal gland serves as the HPA axis’ primary target during stress responses. This gland mediates the secretion of glucocorticoids, hormones that are essential for energy metabolism and stress adaptation. When the HPA axis is activated in chicken, corticotropin-releasing hormone (CRH) is produced by the nucleus of the hippocampal commissure, while arginine vasotocin (AVT) is released from the paraventricular nucleus of the hypothalamus [22, 23]. AVT, a neurohormone unique to chicken, shares structural and functional similarities with arginine vasopressin, which is found in mammals. This similarity highlights a conserved mechanism of stress response across species, albeit with some species-specific adaptations [22].

CRH and AVT act on the anterior pituitary gland, stimulating it to release adrenocorticotropic hormone. This hormone then triggers adrenal cortical cells to release glucocorticoids, primarily cortisol and corticosterone [24]. Glucocorticoids play a dual role: they prepare the organism to cope with stress by mobilizing energy reserves and subsequently exert negative feedback on the hypothalamus. This feedback mechanism ensures that the HPA axis returns to baseline activity, preventing overactivation and protecting against potential disruptions to homeostasis [16, 25]. This self-regulatory loop is a cornerstone of stress physiology, ensuring an optimal balance between the organism’s immediate and long-term needs.

## Effect of heat stress on poultry

Among the consequences of this climatic temperature change is the exacerbation of heat stress in poultry, a critical concern given their narrow thermoneutral zones for optimal productivity 19–22 °C for layers and 18–22 °C for broilers. Temperatures exceeding these ranges impose thermal stress, manifesting as reduced feed intake, slower growth rates, and decreased fertility [2]. Elevated temperatures beyond 32 °C have been shown to significantly decrease feed intake and body weight in broilers, highlighting the direct impact of environmental temperature on poultry growth [26].

### Heat stress on physiological response

Elevated environmental temperatures challenge the thermoregulatory capabilities of chickens, leading to an imbalance between metabolic heat production and the capacity for heat dissipation. Chickens lack sweat glands and rely primarily on latent heat loss through respiration, as their limited unfathered body areas reduce the efficacy of sensible heat loss via conduction, radiation, and convection [7]. As temperatures rise, the thermal gradient between the chicken’s body surface and its environment decreases, making heat dissipation less effective. Consequently, chickens increase their respiratory rate, a process known as thermal tachypnea or panting, to enhance latent heat loss through evaporative cooling from the respiratory tract.

However, the effectiveness of this cooling mechanism is constrained by relative humidity, which limits evaporation and, consequently, heat dissipation [7]. With persistent high temperatures and humidity, the capacity to remove heat is significantly reduced, exacerbating the harmful effects of heat stress on chickens, including elevated risks of hyperthermia [27, 28]. Extended periods of heat stress led to changes in breathing patterns, shifting from rapid panting to slower, deeper breaths known as thermal hyperpnea [7]. This increased respiratory activity can cause hypocapnia and subsequent respiratory alkalosis, complicating the acid–base balance and posing a threat to broiler health [3, 7, 29].

The physiological strain from heat stress also leads to behaviors aimed at increasing heat dissipation, such as lifting the wings to expose less feathered body areas. These behaviors, while crucial for maintaining thermal balance, are energy-intensive and divert calories away from growth and productivity [30, 31]. The resultant lethargy from heat stress often leads to decreased feeding and activity levels, further impacting on the productive performance of chickens, traditionally evidenced by a reduction in feed intake and altered carcass composition, notably a decrease in lean tissue and breast yield [32, 33].

Moreover, heat stress triggers the HPA axis, elevating levels of corticosterone, which has been linked to decreased growth potential, increased proteolysis, suppressed protein synthesis in skeletal muscles, and enhanced fat deposition [29, 34]. This hormonal response further impairs muscle protein metabolism through changes in *S6K1* and *FBXO32* expression. *S6K1*, a downstream effector of the mTOR pathway, promotes muscle protein synthesis but is suppressed during heat stress, reducing protein accretion. And *FBXO32*, which is a muscle-specific E3 ubiquitin ligase, is upregulated, accelerating proteasomal protein degradation. Together, these changes impair muscle protein metabolism and contribute to growth suppression in heat-stressed chickens. These interconnected responses highlight the multifaceted impact of heat stress on chickens, affecting their behavior, physiology, welfare, and overall productivity.

### Heat stress on production performance

Chronic heat stress significantly impacts broiler chickens and laying hens, affecting their growth, production performance, and meat and egg quality in various ways. Sohail et al. found that broilers subjected to chronic heat stress (35 ± 2 °C, continuously from day 1 to day 42) exhibit notable declines in feed intake by 16.4% and body weight by 32.6%, along with an increased feed conversion ratio of 25.6% by the age of 42 d [35]. These adverse conditions also lead to altered meat quality, with a breed-dependent variation in fat deposition reported. Additionally, another investigation found a shift in muscle distribution under heat stress conditions, with a decrease in breast muscle and an increase in thigh muscle proportions; this study also noted a decrease in protein content and an increase in fat deposition [36]. This shift may be attributed to the greater susceptibility of the pectoralis major to reduced protein synthesis and deposition under chronic high temperature, as heat stress lowers ribosomal capacity and decreases muscle protein accretion [37, 38]. Furthermore, the detrimental effects of heat stress extend to egg production in laying hens. One study observed a 28.8% reduction in egg production following a 12-d period of heat stress, accompanied by a significant daily reduction in feed intake [39]. Similarly, other research highlighted that heat stress leads to decreased production performance, thinner eggshells, and increased egg breakage [40], along with significant reductions in egg weight, eggshell thickness, eggshell weight, and eggshell percentage [41]. These findings are corroborated by previous study, where also reported decreased egg production, egg weight, and eggshell thickness due to heat stress [42]. In the broader context of animal husbandry, heat stress not only hampers growth performance but also disrupts organ and muscle metabolism and fat deposition. This disturbance in the balance of energy among fats, carbohydrates, and proteins results in decreased meat quality, increasing the production of lower quality meat types such as pale, soft, and exudative or dark, firm, and dry meat [43, 44]. Overall, the compilation of these studies underscores the increasing adverse effects that chronic heat stress imposes on poultry, ranging from growth inhibition and metabolic disruptions to reduced product quality, thereby impacting overall production efficiency in the livestock industry.

### Heat stress on immune response

The avian immune system includes both innate immunities, serving as the initial defense, and adaptive immunity, involving T and B cells that mediate cellular and humoral responses to antigens, respectively [16, 45]. B cells produce antibodies, and the three types of antibodies found in chicken are IgM, IgA and IgG. Heat stress can reduce the immune response in poultry by decreasing the size and functionality of immune organs like the spleen, thymus, and bursa. This shrinkage occurs because heat stress elevates circulating glucocorticoids, which suppress lymphocyte proliferation, promote apoptosis, and cause morphological damage in lymphoid tissues such as the thymus and bursa, leading to atrophy [16, 45, 46]. This reduction in organ function can lead to increased susceptibility to infections and diminished overall health in affected chicken. Consequently, the incidence of infectious poultry diseases like Newcastle disease tends to be higher during the summer in tropical regions [47]. Heat stress leading to increased growth and colonization of harmful bacteria in the crop and intestines, which induces morphological alterations in intestinal lymphoid cells [48]. These pathogens are then presented to immune cells through the antigen-presenting system, triggering the activation of pro-inflammatory cytokines to combat the invaders [28, 48]. Ultimately, heat stress modifies the body's immune response to pathogens by altering Toll-like receptors and pro-inflammatory cytokines in poultry.

### Heat stress on alteration of blood biochemistry

Heat stress in chickens triggers a series of physiological adjustments impacting blood biochemistry leading to respiratory alkalosis. As chickens engage in mechanisms like panting and gular flutter to shed excess heat, there is an increased expulsion of CO_2_, which subsequently reduces blood bicarbonate levels and elevates plasma pH [49]. This shift is associated with a decrease in circulating potassium (K^+^), likely exacerbated by potassium loss through urine and hemodilution stemming from elevated water intake. These biochemical changes are crucial as they contribute to the observed mortality rates under heat stress conditions. Further, complicating this scenario is the activation of the HPA axis, which leads to heightened levels of the primary stress hormone, corticosterone (CORT). This elevation in CORT is correlated with an increase in blood glucose levels, a response likely driven by enhanced hepatic gluconeogenesis, where lactate is partially converted to glucose [50, 51]. Such metabolic adjustments are part of the broader fight-or-flight response that is vital for survival under stress, significant changes in blood metabolites, including glucose, in broiler breeders exposed to temperatures above 33 °C [52].

Additionally, heat stress influences thyroid hormone levels, which plays a critical role in metabolic regulation and heat production. While heat stress was found to increase thyroxine levels, triiodothyronine levels were significantly reduced, indicating a decrease in the hepatic conversion, potentially due to altered activity of type I iodothyronine deiodinase [26]. The dysregulation of thyroid hormones under high ambient temperatures can be seen as a protective mechanism, moderating the metabolic rate and reducing heat production to conserve energy and enhance survival under thermal stress.

## Expression of different genes during heat stress conditions

Gene expression analysis during heat stress reveals how poultry adapts to thermal challenges. Some genes are upregulated to enhance stress responses and antioxidant defenses, while others are downregulated to conserve energy. Heat stress-responsive genes can be organized into functional hierarchies reflecting their relative importance in adaptation. First, cellular defense is mediated by heat shock proteins (*HSP70*,* HSP90*,* HSP27*), which prevent protein misfolding and aggregation. Second, antioxidant factors such as *SOD*, *CAT*, and *SESN1* detoxify reactive oxygen species, forming the second layer of protection. Third, metabolic and energy regulation genes (*ANGPTL4*,* PLCB4*,* DIO3*) adjust lipid metabolism and thyroid hormone activity to sustain energy balance. Fourth, immune-modulating genes including *IL15*,* TLR4*, and *NFKB1* coordinate inflammatory responses. This categorical framework highlights the progressive organization of stress adaptation mechanisms, from immediate cytoprotection to long-term systemic regulation. While these hierarchies outline the relative functional importance of stress-responsive genes, their expression is not uniform. Instead, it is strongly influenced by external environmental conditions that shape the magnitude and duration of the genetic response.

Environmental factors such as humidity and the duration of heat exposure (acute vs. chronic) critically modulate gene expression responses in poultry. Elevated humidity exacerbates thermal stress by reducing the efficiency of evaporative cooling through panting, thereby intensifying hyperthermia. Under such conditions, the expression of heat shock proteins (*HSP70*,* HSP90*) and oxidative stress-related genes (*SOD*,* CAT*) is often amplified, reflecting an increased demand for cytoprotective mechanisms and antioxidant defenses [53, 54].

The duration of heat exposure also shapes transcriptional responses. Chronic heat stress promotes stable, long-term adaptation through enhanced expression of molecular chaperones and antioxidant pathways. For instance, in the retina, chronic heat stress induced sustained upregulation of *HSP27*,* HSP60*,* HSP70*, and *HSP90* across developmental stages [55]. In the heart, chronic stress caused stronger induction of *NRF2* and *CAT* (antioxidant factors) and more consistent modulation of inflammatory mediators (*NFKB1*,* LITAF*), compared to acute stress, which triggered rapid but less sustained inflammatory responses [56]. Transcriptome studies in the heart and liver further confirmed that chronic stress induces broader reprogramming (2,503 and 2,236 DEGs, respectively) compared to acute stress (1,217 and 1,843 DEGs, respectively), with conserved upregulation of HSP genes (*HSP90AA1*,* HSPA4*,* HSPB8*) and antioxidant genes (*GPX1*), while tissue-specific adaptations involved immune signaling in the heart and metabolic remodeling in the liver [57]. Collectively, these findings highlight that environmental factors such as humidity and the duration of heat exposure fundamentally shape transcriptional responses across multiple tissues. An overview of gene expression changes in response to heat stress across different poultry tissues is provided in Table 1.

**Table 1:** Gene expression in response to heat stress in poultry

No.	Heat stress-related genes	Expression pattern	Animal	Tissue	Function of genes	Ref.
1	*HSP27* (small *HSP*), *HSP70*, and *HSP90*	Upregulated	Broiler	Bursa of Fabricius, and spleen	Prevents protein aggregation, stabilizes signaling proteins under stress	[58]
2	*HSP25*, *HSP70*, *HSP90*, *CAT*, *SOD*, *IL-4* and *IL17*	Upregulated	Broiler	Spleen	Inflammatory and oxidative mediators	[59]
3	*HSP70*	Upregulated	Layer	Serum	Protein folding, prevents aggregation, refolds misfolded proteins	[60]
4	*DPEP2*,* KCNK4*,* CRISPLD2*	Upregulated	Layer	Bursa	Modulating inflammatory responses, maintaining homeostasis, and protecting against cellular damage to enhance stress adaptation	[61]
5	*HSP60*,* HSP70*, and *HSP90*	Upregulated	Broiler	Heart	Protein folding, translocation, and stress response	[62]
6	*HSP70*,* SOD*	Upregulated	Broiler	Liver	Protein folding, supports protein transport, protects cells by converting harmful superoxide radicals into oxygen and hydrogen peroxide	[63]
7	*CCK*,* DIO3*,* BRCC3*, and *FGF14*	Upregulated	Broiler	Liver	Reduces digestion heat, lowers metabolism, repairs DNA, and protects neurons during heat stress	[64]
8	*HSP47 *and *HSP60*	Upregulated	Broiler	Kidney	*HSP47* is a collagen-specific chaperone essential for collagen folding and secretion, while *HSP60* assists in protein folding and refolding within mitochondria	[65]
9	*HSP70*,* HSP90AA1*,* HSPB8*,* HSPA5*,* DNAJB6*,* HSPA8*,* HSPB1*, *HSPA4*,* AHSA2*,* FKBP4*, and *ST13*	Upregulated	Rooster	Testis	Enhances protein folding, prevents aggregation, promotes refolding of damaged proteins, and strengthens stress response pathways	[66]
10	*HSP70 *and *HSP90*	Upregulated	Local chicken	Muscle	Protein folding, prevents aggregation, and supports muscle cell survival	[9]
11	*CHST9*,* HSPA8*,* FABP4*,* SESN1*,* CCNB2*,* CRB2*,* NR4A3*	Upregulated	Indigenous chicken	Muscle	Enhance protein folding, regulate fatty acid metabolism, maintain antioxidant defense, control the cell cycle, and promote cell adhesion and transcriptional responses to stress	[4]
12	*SOD*	Upregulated	Broiler	Skeletal muscle	Enhances the antioxidant defense by converting harmful superoxide radicals into less toxic hydrogen peroxide	[67]
13	*ANGPTL4*	Upregulated	Layer	Liver	Enhances lipid metabolism and promotes energy homeostasis to mitigate stress-induced damage	[68]
14	*BMP10* and *MYH7*	Upregulated	Broiler	Heart	Promotes cardiac growth and maturation, increases slow-contracting myosin heavy chains, potentially contributing to compensatory cardiac hypertrophy	[69]
15	*HSP*,* MYLK2*, and *BDKRB1*	Upregulated	Broiler	Blood	Enhance protein protection, muscle contraction, and inflammatory response, promoting cellular protection	[15]
16	*CNTFR*,* FURIN*,* CCR6*,* LIFR* and *IL20RA*	Upregulated	Layer	Heart, liver, spleen, lung and kidney	Promotes immune modulation, inflammation control, and cellular survival mechanisms to enhance stress resilience and tissue repair	[70]
17	*HSP27 *and *HSP90*	Downregulated	Broiler	Thymus	Prevents protein aggregation, stabilizes cytoskeleton, stabilizes signaling proteins	[58]
18	*CYP3A80 *and *CIRBP*	Downregulated	Rooster	Testis	Reduce detoxification activity and impair cold-induced RNA-binding	[66]
19	*GH*	Downregulated	Broilers	Liver	Stimulates growth, cell reproduction, and regeneration	[63]
20	*CASP6*	Downregulated	Broiler	Liver	Reduced activation of apoptosis	[71]
21	*CRHR1, MEOX1,* and *MOV10L1*	Downregulated	Layer	Bone marrow cell	Affected the intensity and duration of inflammation when experiencing synergistic stimulation	[72]
22	*CCR6 *and *LIFR*	Downregulated	Layer	Heart, liver, spleen, lung and kidney	Weakens immune response and disrupts cellular survival signaling, reducing the ability to combat stress and inflammation	[70]
23	*TRPC5*,* DIO2*, and *ANGPTL4*	Downregulated	Broiler	Liver	Regulates calcium signaling, activates thyroid hormones to increase metabolism, and controls lipid metabolism and vascular development	[64]
24	*CYHR1*,* WNT6*,* WNT10A*	Downregulated	Layer	Bursa	Hinders cell proliferation, tissue repair, and developmental signaling pathways, impairing growth and regenerative processes	[61]
25	*MYH1E* and* XKR9*	Downregulated	Broiler	Heart	Reduced fast-contracting muscle fiber activity and reflect decreased involvement in cell membrane repair or apoptosis regulation	[69]
26	*OCLN*,* CLDN1*,* CLDN4*,* TJP1*,* MUC2*	Downregulated	Broiler	Duodenum, jejunum, ileum	Impairs intestinal barrier integrity and mucus production, leading to increased gut permeability and compromised gut health	[73]
27	*HSPA5*,* SSR1*,* SDF2L1*,* SEC23B*	Downregulated	Local chicken	Liver	Disrupts protein folding, processing, and secretion, impairing endoplasmic reticulum function and cellular stress response	[74]

### Genes related to production

In commercial poultry farming, optimizing high production levels has traditionally been a priority, which has subsequently increased the vulnerability of broilers to environmental stressors. Under high temperature conditions, even breeds that typically exhibit significant weight gain in thermo-neutral environments struggle to sustain their growth performance, leading to decreased productivity [75]. One of the critical areas affected by heat stress is the development of skeletal muscle, which is regulated by myogenic regulatory factors (MRFs). Studies have consistently shown that heat stress adversely affects these factors, with observed reductions in the expression levels of *MYOD1* and *MYOG* in chicken embryos exposed to elevated temperatures [76]. Muscle fiber number in chickens is determined at birth and does not increase; growth is achieved through hypertrophy, primarily by the deposition of proteins which enlarge the existing fibers [77]. However, heat stress disrupts this process of muscle hypertrophy. It diminishes the expression levels of the *IGF1* gene and the concentration of circulating IGF-1, crucial for normal muscle development. The impairment extends to a reduction in the expression of *MYOD* and *MYOG*, further inhibiting muscle hypertrophy by affecting the *S6K1* pathway, a key regulator of cell growth and muscle development [76, 78]. Additionally, heat stress impacts the mRNA expressions of *IGF1* and its downstream genes in the breast muscle, leading to the inactivity of the mechanistic target of rapamycin (*MTOR*) pathway and its target *S6K1*, which are essential for regulating myogenic regulatory factors (*MYOD1*,* MYOG*,* MYF5*,* MYF6*) and facilitating muscle hypertrophy. This inactivity is compounded by a decrease in muscle protein synthesis, attributed to reduced amino acid uptake and the expressions of specific transporter isoforms. Such reductions are directly linked to the compromised functionality of *MTOR* and *S6K1*, illustrating the extensive impact of heat stress on the molecular mechanisms underlying muscle growth in poultry [76].

### Genes related to reproduction

Heat stress is a critical factor negatively impacting the reproductive performance of laying and breeder hens. Multiple studies have documented the detrimental effects of elevated temperatures on ovulation rates, fertility, and hatchability in poultry. Specifically, heat stress has been shown to reduce the ovulation rate, thereby diminishing overall reproductive performance [41, 79]. Fertility and hatchability rates are adversely affected, resulting in significant reproductive challenges [80]. The underlying mechanisms involve the impairment of follicular and oocyte development and a decreased yolk maturation rate, leading to infertility issues in poultry [81, 82]. These reproductive dysfunctions are thought to be associated with a reduction in the secretion of key reproductive hormones such as gonadotropin-releasing hormone (GnRH), luteinizing hormone (LH), and follicle-stimulating hormone (FSH), along with alterations in HSP, fatty acid composition, and antioxidant levels [79].

In addition, heat stress also alters the expression of genes involved in steroidogenesis and folliculogenesis (such as *STAR*,* CYP19A1*,* LHR*,* FSHR*). Yan et al. investigated heat-stressed laying hens and found that *STAR*, *CYP11A1*, and *3βHSD* expression in granulosa cells increased initially, while *FSHR* and *CYP19A1* expression decreased under heat stress conditions [83, 84]. *STAR* and *CYP11A1* are key regulators of steroidogenesis, mediating cholesterol transport into mitochondria and its conversion to pregnenolone. *HSD3B* catalyzes the conversion of pregnenolone to progesterone, while *CYP19A1* encodes aromatase, which converts androgens to estrogens. *FSHR* and *LHR* are gonadotropin receptors that mediate follicular growth and ovulation in response to pituitary hormones. Microarray studies have revealed that heat stress leads to the upregulation of several HSP genes, including *HSP70*, *HSP90AA1*, and *HSP25* in chicken testes [66]. In agreement with this, Wang et al. [85] reported that acute heat exposure (4 h) in broiler-type Taiwanese chickens induced the expression of multiple HSP family members (*HSP70*,* HSP25*,* HSP90AA1*,* HSPA8*,* HSPA5*,* HSPH1*, and *HSPD1*) in testicular tissue. Consistently, elevated *HSP70* expression has also been observed in granulosa cells of heat-stressed laying hens, indicating activation of stress-response pathways that may protect ovarian function [83]. Exposure to acute heat burden also disrupts the hypothalamic regulation of reproductive functions in laying hens, resulting in lower circulating levels of LH. This disruption is primarily due to the reduced functionality of the hypothalamus under heat stress conditions [82]. Interestingly, it has been observed that breeder hens inseminated during the cooler morning hours exhibit higher fertility and hatchability compared to those inseminated during the hotter afternoon hours [86]. This suggests that timing of insemination can mitigate some negative effects of heat stress.

Furthermore, heat stress induces oxidative damage to the small yellow follicles, ovaries, and oviducts in laying hens, ducks, and quails. This occurs primarily through mitochondrial overproduction of reactive oxygen species (ROS), coupled with reduced activity of antioxidant enzymes such as SOD, CAT, and GPx. The resulting oxidative stress promotes lipid peroxidation, protein carbonylation, and DNA damage in reproductive tissues, thereby impairing follicular development and oviductal function. This oxidative damage significantly reduces the relative weights of these reproductive organs and the number of large follicles, leading to decreased egg production performance. In severe cases, such oxidative stress can result in infertility [76, 87, 88].

In male breeders, the repercussions of heat stress are even more pronounced compared to female breeders. Temperatures beyond the thermoneutral zone trigger lipid peroxidation, causing damage to the testis and adversely affecting seminal parameters in male Japanese quail and broiler chickens [89–91]. Seminal parameters, including semen production, sperm metabolism, quality, and motility, are significantly influenced by factors such as temperature, pH, and ion concentration. These factors ultimately lead to infertility and the production of poor-quality spermatozoa [92–94]. During the early phases of heat stress, there may be an initial increase in testicular growth, semen volume, and concentration; however, continued exposure to high temperatures eventually suppresses reproductive capacity in poultry [86]. Research has shown that reproductive efficiency is significantly reduced in males of five different poultry breeds when exposed to heat stress, as evidenced by decreased semen quality and quantity beyond the zone of thermal comfort. Additionally, hens inseminated with semen collected from heat-stressed roosters exhibit a reduced percentage of fertilized eggs due to a decline in sperm-egg penetration capability. Heat stress poses a significant threat to the reproductive performance of both female and male poultry. It affects hormone secretion, damages reproductive organs, and compromises the quality and functionality of gametes. Understanding and mitigating the impacts of heat stress is crucial for maintaining high reproductive efficiency and productivity in poultry farming.

### Expression of immune system genes during heat stress

The immune system is important for poultry due to its direct impact on production performance. Cytokines and Toll-like receptors (TLRs) are indeed critical components of the immune system. Cytokines are pivotal in immune regulation, functioning through hematopoietic cells to aid in host defense and maintain homeostasis. These molecules are diverse, primarily comprising interferons (IFNs), interleukins (ILs), transforming growth factors (TGFs), tumor necrosis factors (TNFs), and chemokines. Each of these cytokines has specific functions in regulating the immune response, ensuring the body can effectively respond to infections and other immune challenges [95]. TLRs, on the other hand, recognize pathogen-associated molecular patterns (PAMPs) and facilitate antigen presentation [77]. Heat stress affects the chicken immune system by altering gene expression, potentially due to various underlying mechanisms. One important mechanism is the compromised integrity of the intestinal barrier. Under heat stress, redistribution of blood flow to the skin reduces intestinal perfusion, resulting in local hypoxia and oxidative stress [96]. This condition disrupts epithelial tight junction proteins such as occludin, claudins, and ZO-1, thereby increasing intestinal permeability (‘leaky gut’) [97]. In addition, excessive production of pro-inflammatory cytokines (e.g., TNF-α, IL-6) and shifts in gut microbiota composition further weaken the epithelial barrier, facilitating the translocation of pathogens and endotoxins across the gut lining. This breakdown of barrier function leads to increased antigen presentation to T cells via TLRs. Consequently, enhanced cytokine and interleukin responses can cause hypervascular permeability, inflammation, and tissue damage [98].

Cytokines, which encode essential immune proteins, act as endogenous signaling molecules in cellular defense against inflammation triggered by high temperatures [99]. ILs are a subset of cytokines, play a critical role in stimulating immune responses and inflammation. They range from IL-1 to IL-17, each with specific functions. Pro-inflammatory cytokines such as IL-1, IL-2, IL-6, IL-18, and TNFα are particularly active in the inflammatory response during heat stress [78]. Their expression increases under high temperatures, likely enhancing immune function by proliferating lymphocytes and macrophages [100, 101]. However, excessive expression and proliferation can lead to tissue damage. Vitamins are known to mitigate the adverse effects of heat stress on growth performance and immunity by suppressing pro-inflammatory cytokine expression, making antioxidant vitamin supplementation advisable in tropical regions [100, 102]. IL-15 is crucial for the growth and proliferation of T-cells, B-cells, intestinal epithelium, and natural killer cells [103]. Recent studies have shown that acute heat stress (40 °C for 7 h) increases *IL15* gene expression in the chicken spleen, suggesting a rapid response to help maintain homeostasis by proliferating immune cells [7].

TLR genes, a conserved group of DNA molecules, play a pivotal role in innate immunity by recognizing PAMPs. TLRs are activated by various components, including antiviral compounds and single-stranded RNAs, and are crucial in the immune response to viruses like influenza during stress [77]. Heat stress affects the gastrointestinal tract, altering the microbiota and intestinal barrier integrity. For instance, broilers exposed to 38–39 °C for 6 h daily for 5 d show increased *TLR4* gene expression in the spleen and intestinal tissues (jejunum and ileum) [101]. This increased expression may result from compromised intestinal health, allowing luminal pathogens to penetrate the intestinal epithelium, thereby activating TLR signaling and causing inflammation. The *TLR4* gene expression is higher in the ileum compared to the jejunum under heat stress, suggesting more severe heat-induced damage in the ileum, possibly due to differences in microbiota composition between these intestinal locations [104].

## Molecular mechanism of heat shock protein activation under heat stress

**Fig. 2:**
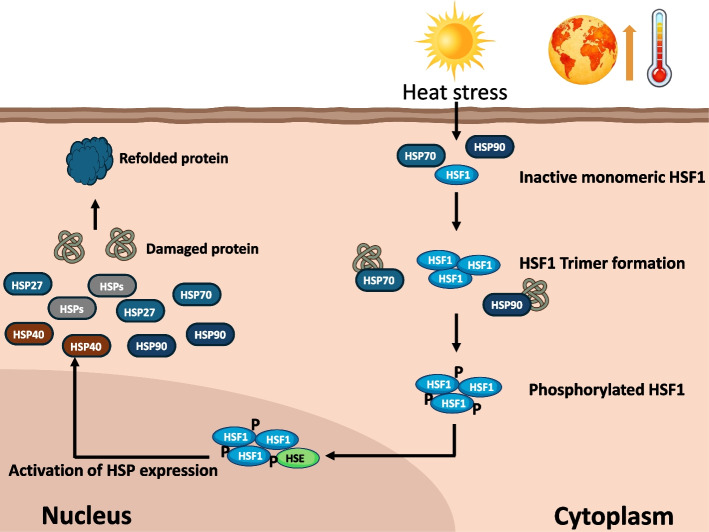
Mechanism of heat shock protein activation during heat stress in chickens

The heat shock response is an essential cellular defense mechanism triggered by thermal stress to protect proteins from misfolding and aggregation [8, 9] (Fig. 2). This figure depicts the molecular cascade that begins when heat stress disrupts protein homeostasis, leading to the accumulation of damaged or misfolded proteins in the cytoplasm. In response to heat stress, inactive monomeric heat shock factor 1 (*HSF1*), bound to *HSP70* and *HSP90* in the cytoplasm, is released and forms trimers [105, 106]. These *HSF1* trimers undergo phosphorylation, enabling their translocation into the nucleus [106]. Once in the nucleus, phosphorylated *HSF1* binds to heat shock elements (HSEs) in the promoter regions of HSP genes, initiating their transcription. Key HSP genes, including *HSP27*,* HSP40*,* HSP70*, and *HSP90*, are then synthesized and perform critical roles in stabilizing cellular functions under heat stress [107]. These proteins act as molecular chaperones to refold damaged proteins, prevent aggregation, and maintain the structural integrity of essential proteins. For example, *HSP70* is involved in binding misfolded proteins and facilitating their proper refolding, while *HSP90* stabilizes signaling proteins that are crucial for cell survival. *HSP40* assists in protein folding and prevents the accumulation of toxic protein aggregates [8, 9, 73]. *HSP27* plays a protective role by stabilizing the cytoskeleton and mitigating stress-induced damage. This coordinated response ensures that cellular processes continue despite thermal stress, reducing the risk of apoptosis or irreversible cellular damage [73, 108]. The efficient activation and function of HSP are vital for maintaining protein homeostasis (proteostasis), particularly in poultry exposed to extreme temperatures. This mechanism not only protects cellular integrity but also improves the organism's ability to adapt to repeated or prolonged heat stress. These molecular processes are critical for developing strategies to enhance heat stress resilience.

## Heat stress resilience genes in poultry

Heat stress resilience in poultry is a critical aspect of maintaining health and productivity, especially in regions with high ambient temperatures. The ability of poultry to withstand heat stress is largely influenced by genetic factors, particularly through the action of specific genes and the production of *HSP*. The HSPs are crucial stress proteins found in the cells of all living organisms. These proteins are activated in response to high ambient temperatures, initiating a "heat shock response" that protects cells from heat-induced damage [103]. During this response, the production of HSPs increases in stressed cells, which helps in the synthesis and proper folding of other proteins, ensuring cellular function is maintained.

HSPs are categorized into six classes based on their molecular weight: *HSP40*,* HSP70*,* HSP90*, *HSP100*, small *HSP*, and chaperonins. These proteins act as stress signals originating from the extracellular environment and play a significant role in triggering immune responses during stress and adverse conditions. Among these, *HSP70* is particularly important for cellular recovery following heat stress damage [8]. The upregulation of HSPs during heat stress is an adaptive mechanism that improves cellular tolerance to heat stress, enhancing the organism's overall resilience. Expression of *RB1CC1*, *BAG3*, and *CITED2* genes increased during heat stress, supporting cell survival by distinct mechanisms [109]. *RB1CC1* promotes autophagy and clearance of damaged proteins, thereby reducing proteotoxic stress. *BAG3* acts as a co-chaperone with *HSP70* to stabilize cytoskeletal proteins, facilitate removal of misfolded proteins, and inhibit apoptosis [110]. *CITED2* functions as a transcriptional co-regulator, modulating hypoxia-inducible factor 1 (HIF1) and CBP/p300 pathways to fine-tune stress-responsive transcription and suppress excessive apoptotic signaling [111]. Together, the upregulation of these genes contributes to enhanced cellular tolerance and resilience under heat stress. Certain HSP genes, such as *HSP40, HSP70,* and *HSP90*, are upregulated to stabilize and refold denatured proteins, a critical process for cell survival under heat stress [9]. The expression of the *TRMT1L* gene is elevated, which is essential for maintaining redox homeostasis, thereby supporting proper cellular proliferation and survival under oxidative stress [112, 113]. Additionally, the *NFAT5* and *NFKB1* genes are upregulated to stimulate the expression of various pro-inflammatory cytokines [114]. The *PLCB4* gene shows an increase in expression that plays a crucial role in regulating metabolic energy processes, enhancing the organism's ability to manage energy efficiently during stress conditions [115]. Similarly, the genes *H1F0* and *ACYP* are elevated to counteract the effects of heat stress. These increases are essential for reducing heat-induced apoptosis and promoting DNA repair mechanisms, thereby protecting cellular integrity under stressful conditions [4].

Breed-specific variation in the expression of heat stress-responsive genes has been extensively documented. Indigenous chickens such as the Fayoumi exhibit consistently higher *HSP70* expression and stronger antioxidant responses than commercial lines, reflecting their superior resilience [68]. In Egyptian breeds, Dandarawi and Sinai chickens showed markedly elevated expression of *HSP70* and *CPT1*, with the naked neck gene further enhancing *HSP70* expression and metabolic adaptation [116, 117]. Likewise, Sard et al. [118] reported that indigenous Isfahan chickens displayed stronger induction of heat shock proteins, whereas commercial Ross broilers exhibited greater upregulation of innate immune genes such as *TLR4* and *IL4I1*, indicating divergent genetic strategies for coping with thermal stress. In contrast, commercial broilers often depend on the induction of *HSP70*, *HSP90*, and metabolic regulators under chronic heat stress but generally demonstrate a less efficient adaptive capacity compared to indigenous lines [9, 64]. Collectively, these findings confirm that resilience genes—including *HSP*s, antioxidant enzymes, *CPT1*, and immune regulators—have been validated across diverse indigenous and commercial breeds, underscoring their importance as candidate markers for genetic improvement programs. Recent transcriptomic studies further support this evidence: in Kenyan indigenous chickens from lowland (Mombasa) and highland (Naivasha) ecotypes, qRT-PCR validated the expression of *HSPH1*, *PDK4*, and *SRGN* [4], while in the L2 strain of Taiwan country chickens, acute heat stress significantly upregulated resilience-associated genes such as *HSP70*, *HSP90AA1, HSP25, BAG3*, and *DNAJA4* [66]. These genes play critical roles in protein folding, apoptosis regulation, and cellular protection, highlighting their potential as candidate markers for thermotolerance in indigenous breeds.

## Genetic adaptations for heat tolerance in chickens

In broiler chickens, genetic selection for heat tolerance is especially crucial in tropical and subtropical regions where high temperatures are common. The "frizzled feather" phenotype, which features outwardly curled feathers, was first documented by Darwin and is associated with better heat dissipation. Key genes such as naked neck (*Na*), frizzle (*F*, with candidate genes *KRT6A* and *KRT75L4*), and dwarf (*Dw*, with the candidate gene *GHR*) have been identified as contributors to thermal stress tolerance in poultry.

The *Na* gene significantly reduces feather mass by up to 40%, which enhances heat dissipation by minimizing insulation from feathers [119]. Birds with this gene exhibit improved immunity and production performance, likely due to better thermoregulation and reduced metabolic stress [80]. Additionally, the *Na* gene plays a role in minimizing fat deposition in the breast region, further promoting heat tolerance [120–123]. The *Dw* is associated with a reduction in body size by 30% to 40%, which is beneficial for heat tolerance as smaller body mass helps in more efficient heat dissipation. Similarly, the *F* contributes to heat tolerance through the altered feather structure, enhancing airflow and cooling [121]. These genetic traits are beneficial for the commercial poultry industry, particularly in regions prone to high temperatures, by improving the overall heat resilience and productivity of poultry. Integrating these genetic markers into breeding programs could significantly enhance the sustainability and efficiency of poultry production in hot climates, ultimately contributing to food security and economic stability in affected regions.

## Future perspectives

Chicken is widely regarded as a cornerstone of global food security due to their adaptability and resilience to climate change. Future initiatives should focus on identifying and characterizing indigenous chicken breeds that are adaptable to diverse agro-ecological zones, using advanced biotechnological tools to screen for climate resilience. Additionally, there is a pressing need for detailed studies to discover permanent genetic markers to assess the adaptive and productive capacities of these breeds. The subsequent phase involves refining promising chicken breeds through marker-assisted breeding to develop breeds that are not only resilient but also optimized for productivity in adverse conditions, thus maximizing the economic returns of poultry enterprises.

Despite these advances, significant challenges remain in translating transcriptomic and genomic insights into practical breeding applications. Current limitations include variability in transcriptomic responses across breeds and environments, difficulties in validating candidate genes within large-scale breeding populations, and the lack of standardized phenotyping protocols for heat resilience traits. These gaps restrict the reliability and reproducibility of genomic selection under field conditions, emphasizing the need for further methodological refinement.

At the same time, targeted gene incorporation strategies, such as the introduction of the *Fa* and *Na* alleles, offer important opportunities to enhance thermotolerance. When combined with nutritional interventions, such genetic strategies have the potential to minimize the adverse effects of elevated temperatures and support poultry adaptation across both temperate and tropical regions. Overcoming current barriers will require the integration of advanced approaches such as marker-assisted selection, genome-wide association studies (GWAS), and multi-omics frameworks. These tools will allow for a more comprehensive understanding of the genetic architecture underlying thermotolerance and facilitate the development of resilient, high-performing poultry breeds suited to future climatic challenges.

## Conclusion

Heat stress poses a significant threat to poultry production, impacting growth, reproduction, immunity, and overall productivity. The molecular insights into heat stress responses, particularly the role of HSP and other stress-regulating genes, highlight the intricate mechanisms that help chickens adapt to thermal challenges. Key genes like *HSP70*, *HSP90*, and *SOD* enhance cellular protection and repair under heat stress, while genetic traits such as the *Na* and *F* genes offer natural thermotolerance. These findings underscore the importance of integrating genetic, transcriptomic, and physiological strategies to develop resilient poultry breeds. Future research should focus on leveraging indigenous breeds, marker-assisted selection, and advanced biotechnological tools to optimize poultry performance in hot climates. Strengthening the heat stress resilience of poultry is essential not only for sustainable production but also for securing sustainable agriculture under climate change.

## Data Availability

Not applicable. This review article is based on previously published literature and does not include any new datasets generated or analyzed by the authors.

## Abbreviations

*ACYP*: Acylphosphatase
*ANGPTL4*: Angiopoietin-like 4
AVT: Arginine vasotocin
*BAG3*: BCL2 associated athanogene 3
*BMP10*: Bone morphogenetic protein 10
*CASP6*: Caspase 6
CAT: Catalase
CCK: Cholecystokinin
*CCNB2*: Cyclin B2
CORT: Corticosterone
CRH: Corticotropin-releasing hormone
*CRHR1*: Corticotropin-releasing hormone receptor 1
CYP11A1: Cytochrome P450 family 11 subfamily A member 1
*CYP3A80*: Cytochrome P450 family 3 subfamily A polypeptide 80
*DIO2/DIO3*: Deiodinase type 2/type 3
DNA: Deoxyribonucleic acid
*Dw*: Dwarf gene
ER: Endoplasmic reticulum
*F*: Frizzle gene
*FBXO32*: F-box protein 32
*FGF14*: Fibroblast growth factor 14
FSH: Follicle-stimulating hormone
*FSHR*: Follicle-stimulating hormone receptor
*GHR*: Growth hormone receptor
GnRH: Gonadotropin-releasing hormone
GPX/GPx: Glutathione peroxidase
GWAS: Genome-wide association studies
*H1F0*: H1 Histone family member 0
*HIF1*: Hypoxia-inducible factor 1
HPA: Hypothalamic-pituitary-adrenal
HSE: Heat shock element
*HSF1*: Heat shock factor 1
*HSP*: Heat shock protein
*HSP100*: Heat shock protein 100
*HSP27*: Heat shock protein 27
*HSP40*: Heat shock protein 40
*HSP70*: Heat shock protein 70
*HSP90*: Heat shock protein 90
IFN: Interferon
Ig: Immunoglobulin
*IGF1*: Insulin-like growth factor 1
IL: Interleukin
*KRT6A*: Keratin 6A
*KRT75L4*: Keratin 75-like 4
LH: Luteinizing hormone
*LHR*: Luteinizing hormone receptor
*MRF*: Myogenic regulatory factor
mRNA: Messenger ribonucleic acid
mTOR: Mechanistic target of rapamycin
*MYH1E*: Myosin heavy chain 1E
*MYH7*: Myosin heavy chain 7
*MYLK2*: Myosin light chain kinase 2
MyoD: Myogenic differentiation 1
MyoG: Myogenin
*Na*: Naked neck gene
*NFAT5*: Nuclear factor of activated t cells 5
*NFκB*: Nuclear factor kappa-light-chain-enhancer of activated B cells
NRF2: Nuclear factor erythroid 2-related factor 2
PAMP: Pathogen-associated molecular pattern
PCR: Polymerase chain reaction
*PDK4*: Pyruvate dehydrogenase kinase 4
*PLCB4*: Phospholipase C beta 4
*RB1CC1*: RB1-inducible coiled-coil 1
RNA: Ribonucleic acid
ROS: Reactive oxygen species
S6K1: Ribosomal protein S6 kinase 1
SAM: Sympatho-adrenal medullary
*SESN1*: Sestrin 1
*STAR*: Steroidogenic acute regulatory protein
TGF: Transforming growth factor
*TLR*: Toll-like receptor
TNFα: Tumor necrosis factor alpha
*TRMT1L*: tRNA methyltransferase 1L
